# Detection of macrolide and disinfectant resistance genes in clinical *Staphylococcus aureus *and coagulase-negative staphylococci

**DOI:** 10.1186/1756-0500-4-453

**Published:** 2011-10-27

**Authors:** Tarek Zmantar, Bochra Kouidhi, Hanene Miladi, Amina Bakhrouf

**Affiliations:** 1 Laboratoire d'Analyse, Traitement et Valorisation des Polluants de l'Environnement et des Produits, Faculté de Pharmacie, Monastir, Tunisia

**Keywords:** *Staphylococcus aureus*, Coagulase-negative Staphylococci, Multidrug resistance, *qac*

## Abstract

**Background:**

*Staphylococcus aureus *and Coagulase-negative staphylococci (CoNS) are a major source of infections associated with indwelling medical devices. Many antiseptic agents are used in hygienic handwash to prevent nosocomial infections by Staphylococci. Our aim was to determine the antibiotic susceptibility and resistance to quaternary ammonium compound of 46 *S. aureus *strains and 71 CoNS.

**Methods:**

*S. aureus *(n = 46) isolated from auricular infection and CoNS (n = 71), 22 of the strains isolated from dialysis fluids and 49 of the strains isolated from needles cultures were investigated. Erythromycin resistance genes (*erm*A, *erm*B, *erm*C, *msr*A and *mef*) were analysed by multiplex PCR and disinfectant-resistant genes (*qac*A, *qac*B, and *qac*C) were studied by PCR-RFLP.

**Results:**

The frequency of erythromycin resistance genes in *S. aureus *was: *erm*A+ 7.7%, *erm*B+ 13.7%, *erm*C+ 6% and *msr*A+ 10.2%. In addition, the number of positive isolates in CoNS was respectively *erm*A+ (9.4%), *erm*B+ (11.1%), *erm*C+ (27.4%), and *msr*A+ (41%). The MIC analyses revealed that 88 isolates (74%) were resistant to quaternary ammonium compound-based disinfectant benzalkonium chloride (BC). 56% of the BC-resistant staphylococcus isolates have at least one of the three resistant disinfectants genes (*qac*A, *qac*B and *qac*C). Nine strains (7.7%) among the CoNS species and two *S. aureus *strains (2%) harboured the three-*qac *genes. In addition, the *qac*C were detected in 41 strains.

**Conclusions:**

Multi-resistant strains towards macrolide and disinfectant were recorded. The investigation of antibiotics and antiseptic-resistant CoNS may provide crucial information on the control of nosocomial infections.

## Background

The increasing number of infections caused by oxacillin-resistant staphylococci makes glycopeptide antibiotics an important choice [1]. The significant prevalence of nosocomial infections caused by multi-resistant *S. aureus *and coagulase-negative staphylococci (CoNS) has been documented [2,3]. These species have the ability to survive in medical devices for months [4]. Resistance to erythromycin in staphylococci is usually associated with resistance to other macrolides. Three genes (*erm*A, *erm*B, and *erm*C) encoding methyltransferases responsible of resistance to macrolides, lincosamides and type B streptogramins (MLS_B _phenotype) by modification of the ribosomal target site have been found in staphylococci [5]. The *msr*A gene displays another mechanism of inducible resistance to erythromycin by encoding an ATP-dependent efflux pump [6]. On the other hand, macrolide efflux is affected by a membrane protein encoded by the *mef *gene [7]. Antiseptic agents include various compounds with different chemical structures [8]. The widespread use of quaternary ammonium compounds (QAC) in hospitals actually contributes to the emergence of disinfectant-resistant bacteria [9,10]. Epidemiological data on antiseptic susceptibility and the distribution of resistance genes are both useful for nosocomial infection control. In several staphylococcal species, *qac*-resistant genes have been identified [11-13]. Found in clinical staphylococci (*qac*A, *qac*B, and *qac*C), these genes are generally carried by plasmids [14-16]. Some of these plasmids (pST6, pSK4, and pSK41) contain antibiotic resistance genes encoding resistance to gentamicin, penicillin, kanamycin, and tobramycine [17,18]. Multidrug resistance pumps have been recognized as mediators of a number of commonly used ammonium antiseptics and detergents [19]. The nearly identical *qac*A/B gene is normally harboured by large plasmids [20,21]. The relation between *qac *resistance and penicillin resistance in human clinical staphylococci is quite prevalent [22].

In the present study, we examined the antibiotic susceptibility and resistance to quaternary ammonium compound-based disinfectant benzalkonium chloride (BC) of 46 *S. aureus *strains isolated from auricular infection and 71 CoNS isolated from dialysis fluids and needles cultures. In order to examine the genetic drug resistance mechanisms, erythromycin resistance genes (*erm*A, *erm*B, and *erm*C) and macrolide efflux genes (*msr*A and *mef*) were analysed by PCR multiplex. In addition, quaternary ammonium resistance genes (*qac*A, *qac*B, and *qac*C) were detected by PCR-RFLP.

## Methods

### Biochemical characterization and antimicrobial susceptibility

A total of 117 clinical staphylococcal strains were isolated from Kairouan, in central of Tunisia including 46 *S. aureus *strains isolated from auricular infection and 71 CoNS strains isolated from dialysis fluid and needles from a dialysis service. All strains were identified using the Api ID32 Staph system (bioMérieux, Marcy l'Étoile, France) according to the manufacturer's recommendations and the results were read using an automated microbiological mini-Api (bioMérieux, Marcy l'Étoile, France). Each strain was tested for 18 antibiotics (penicillin, oxacillin, kanamycin, tobramycin, gentamicin, tetracycline, minocyclin, erythromycin, lincomycin, pristinamycin, fosfomycin, nitrofurantoin, pefloxacin, rifampicin, fusidic acid, vancomycin, teicoplanin and cotrimoxazol) using the ATB Staph system (bioMérieux, France) according to the manufacturer's specifications.

### Minimum inhibitory concentration determination

The broth microdilution method was used to determine the minimum inhibitory concentration (MIC) of benzalchonium chloride (BC) (Acros organics, USA) (ranging from 0.5 to 256 μg/ml, by serial twofold dilutions) against the tested strains as recommended by the Clinical and Laboratory Standards Institute (CLSI) [23]. An inoculum of 10^4 ^-10^5 ^cells ml^-1 ^was used. The lowest concentration of antimicrobial agent totally preventing growth after 24 h was taken to be the MIC.

### Multiplex PCR for the detection of macrolide-resistance encoding genes

Macrolide resistance genes *erm*A, *erm*B, *erm*C, *msr*A, and *mef *were examined in all strains using multiplex PCR, as previously described [24,25]. Multiplex PCR assays were performed in 25 μl PCR mixture 1 and mixture 2. Mixture 1 contained a DNA template (50 ng), 100 μM concentrations (each) of the four dNTPs, 1 U of Go *Taq *DNA polymerase (Promega, Lyon, France), 5 μL green Go *Taq *buffer (5X), 25 pM each of forward and reverse primers *erm*A, *erm*C, and *msr*A (Table 1). The PCR mixtures were subjected to thermal cycling (3 min at 96°C, followed by 30 cycles of 30 sec at 95°C for denaturation, 30 sec at 55°C for annealing extension, and extension at 72°C for 2 min). A final elongation at 72°C for 10 min was achieved in a DNA thermal cycler (GenAmp PCR system 9700-Applied Biosystem Int., USA). For mixture 2, the forward and reverse primers (25 pM) of genes *erm*B and *mef *were used. PCR products were analysed in agarose gel (1%) electrophoresis in 1X Tris-borate-EDTA buffer (TBE) at pH 8.3. The amplification products were photographed and their size was determined using a 100 bp molecular size marker (Bio-Rad, France).

**Table 1:** List of primer used for the detection of genes encoding antibiotics and quaternary ammonium compound resistance

Gene	Primer 5'-3'	Product Size pb	Reference
*erm*A	5'-TAT CTT ATC GTT GAG AAG GGA TT-3' 5'-CTA CAC TTG GCT TAG GAT GAA A-3'	139	[22]
*erm*B	5'-CTA TCT GAT TGT TGA AGA AGG ATT-3' 5'-GTT TAC TCT TGG TTT AGG ATG AAA-3'	142	[22]
*erm*C	5'-CTT GTT GAT CAC GAT AAT TTC C-3' 5'-ATC TTT TAG CAA ACC CGT ATT C-3'	190	[22]
*msr*A	5'-TCC AAT CAT AGC ACA AAA TC-3' 5'-AAT TCC CTC TAT TTG GTG GT-3'	163	[22]
*mef*	5'-AGTATCATTAATCACTAGTGC-3' 5'-TTCTTCTGGTACAAAAGTGG-3'	348	[23]
*qac*A, *qac*B	5'-TCCTTTTAATGCTGGCTTATACC-3' 5'-AGCCKTACCTGCTCCAACTA-3'	220	[24]
*qac*C	5'-GGCTTTTCAAAATTTATACCATCCT-3' 5'-ATGCGATGTTCCGAAAATGT-3'	249	[24]

### Detection of *qac*A, *qac*B, and *qac*C genes by PCR-RFLP

Multiplex PCR-RFLP analysis of the *qac*A/B and *qac*C genes was achieved as described by Sekiguchi et al. [26]. For the fragment *qac*A/*qac*B, the forward primer (corresponding to nucleotides 924-946) and the reverse primer (corresponding to nucleotides 1143-1124) produced 220 bp. For the *qac*C gene, the forward primer (nucleotides 73-97) and the reverse primer (nucleotides 321-302) produced 249 bp. PCR was performed in a 25 μl reaction volume containing 50 ng of extracted DNA, 5 μl green Go *Taq *buffer (5X), 200 μM each of deoxynucleoside triphosphates (dNTP), 25 μM each of *qac*A, *qac*B, and *qac*C forward and reverse primers, 1 U of GO *Taq *DNA polymerase (Promega, USA). Each PCR was performed in duplicate. The reaction mixtures were heated to 94°C for 5 min and were then subjected to 30 denaturation cycles at 94°C for 1.5 min, annealing at 56°C for 0.5 min and extension at 72°C for 1.5 min, ending with a final extension at 72°C for 10 min.

Following PCR, the product was digested with 5 U of *Alu*I at 37°C for 90 min (Promega, France). Ten microlitres of treated product were analysed by gel electrophoresis in 3% agarose gel in 1X Tris-borate-EDTA buffer (TBE, pH 8.3). The amplification products were photographed and their size was determined using a 25 bp molecular size marker (Promega, USA). The *qac*B gene was expected to be digested into two fragments: a visible 176 bp fragment and another fragment of 44 bp which is invisible. *Qac*A and *qac*C genes were not expected to be digested [26].

### Statistical analysis

Statistical analysis was performed on SPSS v.13.0 statistics software. Pearson's chi-square^-2 ^test was used to asses inter-group significance. In addition statistical significance was set at P < 0.05.

## Results

### Biotyping and antimicrobial susceptibility

In this study, 46 *S. aureus *strains isolated from auricular infection were identified. In addition 71 CoNS were isolated and were subdivided into eight species: *S. epidermidis *(n = 32) (45%) followed by *S. hominis *(n = 10) (14.1%), *S. haemolyticus *(n = 9) (12.7%), *S. warneri *(n = 5) (7%), *S. simulans *(n = 6) (8.5%), *S. capitis *(n = 4) (5.6%), *S. chromogenes *(n = 3) (4.2%), and *S. equorum *(n = 2) (2.8%).

The results of the antibiotic susceptibility test confirmed the multi-resistance of 117 staphylococcal strains toward the 18 antibiotics mentioned previously. The majority of these strains were resistant to penicillin (91.1%). The isolated strains were also resistant to kanamycin (45.7%), tetracycline (47.8%), erythromycin (37.7%), lincomycin (21.2%), fosfomycin (20.8%), fusidic acid (17.3%), pefloxacin (18.4%), cotrimoxazol (24.3%), teicoplanin (10.5%), gentamicin (13.4%), rifampicin (12.1%), and tobraycin (16.8%).

Oxacillin-resistant phenotype was found in 9 *S. aureus *(7.7%) and 24 CoNS (20.5%) isolated strains. All of the strains were susceptible to pristinamycin, nitrofurantoin, and vancomycin (Table 2 and 3).

**Table 2:** Distribution of disinfectant and macrolide resistance genes in staphylococci

	Samples	*qac*A	*qac*B	*qac*C	BC^b^	*mec*A	*erm*A	*erm*B	*erm*C	*msrA*	Antibiotic
***S. epidermidis***	E10	+	-	+	16	+	-	+	+	+	Oxa^S^, Ery^S^
	E11	-	-	+	8	+	-	+	+	+	Oxa^R^
	E13	-	-	-	4	-	-	-	-	-	Oxa^R^
	E15	-	-	+	8	+	-	+	+	-	Oxa^R^, Ery^R^
	E18	+	-	+	16	+	-	-	+	+	Oxa^S^, Ery^S^
	E20	-	-	-	2	+	-	-	-	+	Ery^R^
	E21	+	-	+	8	+	-	-	+	+	Oxa^R^
	E24	-	-	-	8	+	-	-	+	+	Ery^R^
	E4	-	-	-	4	-	-	-	-	-	Oxa^R^
	E5	-	-	-	4	-	-	-	-	-	Oxa^R^
	E6	-	-	-	2	+	-	+	+	+	Oxa^S^, Ery^S^
	E7	-	-	+	8	-	-	-	-	-	Ery^R^
	E9	-	-	+	16	+	-	+	-	+	Oxa^S^, Ery^S^
	S12	-	-	+	16	-	-	-	-	+	Oxa^S^, Ery^S^
	S15	-	-	+	8	+	+	+	+	+	Oxa^R^, Ery^R^
	S16	-	-	+	8	+	-	+	+	+	Oxa^S^, Ery^S^
	S2	-	-	+	8	+	-	-	+	+	Oxa^S^, Ery^S^
	S21	+	+	+	32	+	+	-	+	+	Oxa^R^
	S22	-	-	-	8	+	-	-	+	+	Oxa^S^, Ery^S^
	S23	-	-	-	2	+	-	-	-	+	Ery^R^
	S25	-	-	+	8	+	-	+	+	+	Oxa^R^
	S26	-	-	+	4	+	-	+	+	+	Oxa^R^, Ery^R^
	S27	-	-	-	2	-	-	-	-	+	Ery^R^
	S33	-	-	-	2	+	+	-	-	-	Oxa^S^, Ery^S^
	S35	-	-	-	2	-	-	-	-	-	Oxa^S^, Ery^S^
	S38	-	-	-	4	+	-	-	-	-	Oxa^S^, Ery^S^
	S40	-	-	+	8	+	-	-	-	+	Oxa^R^, Ery^R^
	S43	-	-	-	2	+	-	-	-	+	Oxa^S^, Ery^S^
	S48	-	-	-	2	+	+	-	+	-	Oxa^R^, Ery^R^
	S56	-	-	-	2	+	-	+	+	+	Oxa^R^, Ery^R^
	S59	-	-	-	4	+	-	-	-	-	Oxa^S^, Ery^S^
	S9	+	+	+	16	+	-	-	+	+	Oxa^S^, Ery^S^
***S. hominis***	E17	-	-	-	16	+	-	-	+	-	Oxa^R^
	E2	-	-	-	2	-	-	-	-	-	Oxa^S^, Ery^S^
	E27	-	-	-	2	-	-	-	-	+	Oxa^S^, Ery^S^
	S18	+	+	+	16	-	+	-	+	+	Oxa^S^, Ery^S^
	S3	-	-	+	8	+	-	-	+	-	Oxa^R^, Ery^R^
	S45	+	-	-	4	+	-	-	-	-	Oxa^R^, Ery^R^
	S50	-	-	-	4	+	-	-	-	+	Ery^R^
	S53	-	-	-	2	+	-	-	-	+	Ery^R^
	S54	-	-	-	2	-	-	-	-	-	Ery^R^
	S57	+	-	+	8	+	-	-	+	-	Oxa^R^
***S. equorum***	E3	+	-	+	8	+	-	-	-	+	Ery^R^
	S6	+	+	+	16	+	-	+	+	+	Oxa^R^, Ery^R^
***S. capitis***	E25	+	+	+	32	-	+	-	+	+	Oxa^S^, Ery^S^
	S1	+	+	-	32	+	+	-	-	+	Oxa^S^, Ery^S^
	S34	+	-	+	32	-	-	-	-	+	Ery^R^
	S49	-	-	-	8	+	-	-	-	+	Oxa^S^, Ery^S^
***S. hemolyticus***	E16	-	-	+	8	+	-	-	-	+	Ery^R^
	E19	+	+	+	16	+	-	-	-	+	Ery^R^
	E22	-	-	+	8	+	-	-	-	+	Ery^R^
	S10	-	-	-	4	+	-	-	-	+	Oxa^R^, Ery^R^
	S13	+	+	-	8	+	-	-	-	+	Oxa^S^, Ery^S^
	S24	+	+	+	16	+	-	-	-	+	Ery^R^
	S39	+	-	+	16	+	+	-	+	-	Oxa^R^, Ery^R^
	S42	+	-	+	16	+	-	+	-	+	Oxa^R^, Ery^R^
	S8	-	-	-	8	+	-	-	-	-	Oxa^R^, Ery^R^

+, presence of the gene -, absence of the gene; Oxa^R^, oxacillin resistance; Oxa^S^, oxacillin susceptible; BC^b ^, MIC of benzalchonium chloride μg/ml.

**Table 3:** Distribution of disinfectant and macrolide resistance genes in staphylococci

Species	Samples	*qac*A	*qac*B	*qac*C	BC^b^	*mec*A	*erm*A	*erm*B	*erm*C	*msrA*	Antibiotic
***S. warnerie***	S11	-	-	+	8	+	-	-	+	+	Oxa^S^, Ery^S^
	S14	-	-	-	2	-	-	-	-	+	Oxa^S^, Ery^S^
	S29	-	+	-	8	-	-	-	-	+	Oxa^S^, Ery^S^
	S37	-	-	+	8	+	-	-	+	+	Oxa^S^, Ery^S^
	S47	-	+	-	4	+	+	+	+	+	Oxa^S^, Ery^S^
***S. chromogene***	S30	+	-	+	16	+	-	-	+	+	Ery^R^
	S32	-	-	-	2	-	-	-	-	+	Oxa^S^, Ery^S^
	S51	-	-	-	2	-	-	-	-	-	Ery^R^
***S. simulans***	E23	-	-	+	4	+	+	-	+	-	Ery^R^
	S19	+	-	+	16	+	-	-	+	-	Oxa^R^, Ery^R^
	S20	-	-	-	4	+	-	-	-	+	Oxa^S^, Ery^S^
	S28	+	+	+	32	+	+	-	+	+	Oxa^R^, Ery^R^
	S36	+	-	-	8	+	-	-	-	-	Oxa^S^, Ery^S^
	S5	+	+	+	16	+	-	-	+	-	Oxa^R^
***S.aureus***	Sa1	*-*	*-*	**+**	8	+	+	+	-	-	Oxa^R^, Ery^R^
	Sa2	*-*	*-*	*-*	4	-	-	-	-	-	Oxa^S^, Ery^S^
	Sa3	*-*	*-*	*-*	8	-	-	+	-	-	Oxa^S^, Ery^S^
	Sa4	*-*	*-*	*-*	4	-	-	-	-	-	Oxa^S^, Ery^S^
	Sa5	**+**	**+**	*-*	8	-	+	-	+	+	Oxa^S^, Ery^S^
	Sa6	*-*	*-*	*-*	8	-	-	-	-	-	Oxa^S^, Ery^S^
	Sa7	*-*	*-*	*-*	4	-	-	-	-	-	Oxa^S^, Ery^S^
	Sa8	*-*	*-*	*-*	8	+	-	+	-	+	Oxa^R^, Ery^R^
	Sa9	*-*	*-*	*-*	2	-	-	-	+	-	Oxa^S^, Ery^S^
	Sa10	***+***	***+***	*-*	8	+	-	+	-	-	Ery^R^
	Sa11	*-*	*-*	*-*	4	+	-	+	-	+	Ery^R^
	Sa12	*-*	*-*	*-*	8	+	-	+	-	+	Ery^R^
	Sa13	*-*	*-*	*-*	8	+	-	+	-	-	Oxa^S^, Ery^S^
	Sa14	*-*	*-*	*-*	4	+	-	-	-	-	Oxa^R^
	Sa15	*-*	*-*	*-*	8	+	+	+	+	+	Oxa^S^, Ery^S^
	Sa16	*-*	*-*	*-*	4	+	-	-	-	-	Oxa^R^
	Sa17	*-*	*-*	*-*	2	-	-	-	-	+	Ery^R^
	Sa18	*-*	*-*	*-*	2	-	+	+	-	-	Oxa^S^, Ery^S^
	Sa19	**+**	*-*	***+***	8	-	-	+	-	-	Oxa^S^, Ery^S^
	Sa20	*+*	**-**	**+**	4	-	+	+	+	+	Oxa^S^, Ery^S^
	Sa21	*-*	*-*	*-*	16	-	-	+	-	-	Oxa^S^, Ery^S^
	Sa22	**+**	**+**	**+**	16	+	-	-	+	-	Oxa^R^, Ery^R^,
	Sa23	***+***	***+***	***+***	16	+	+	+	+	+	Oxa^R^, Ery^R^
	Sa24	*-*	*-*	*-*	4	-	-	-	-	-	Ery^R^
	Sa25	*-*	*-*	*-*	4	-	-	-	-	-	Oxa^S^, Ery^S^
	Sa26	*-*	*-*	*-*	4	+	-	-	-	+	Oxa^R^, Ery^R^
	Sa27	*-*	*-*	*-*	2	+	+	-	-	+	Oxa^R^, Ery^R^
	Sa28	*-*	*-*	*-*	2	+	-	-	-	-	Ery^R^
	Sa29	*-*	*-*	*-*	2	+	-	-	-	-	Oxa^S^, Ery^S^
	Sa30	*-*	*-*	*-*	8	+	+	+	-	-	Oxa^S^, Ery^S^
	Sa31	*-*	*-*	*-*	2	+	-	+	-	-	Ery^R^
	Sa32	*-*	*-*	*-*	2	+	-	+	-	-	Oxa^R^
	Sa33	*-*	*-*	*-*	4	+	-	-	-	-	Ery^R^
	Sa34	*-*	*-*	*-*	8	+	-	-	-	-	Oxa^S^, Ery^S^
	Sa35	*-*	*-*	*-*	4	+	-	-	-	-	Oxa^S^, Ery^S^
	Sa36	*-*	*-*	*-*	2	-	+	-	+	+	Oxa^S^, Ery^S^
	Sa37, Sa38 Sa43, Sa44 Sa45	*-*	*-*	*-*	8 2 8 8 2	-	-	-	-	-	Oxa^S^, Ery^S^
	Sa39, Sa42 Sa41, Sa40	*-*	*-*	*-*	8 16 2 8	+	-	-	-	-	Oxa^S^, Ery^S^
	Sa46	*-*	*-*	*-*	2	+	-	-	-	+	Oxa^S^, Ery^S^

+, presence of the gene -, absence of the gene; Oxa^R^, oxacillin resistance; Oxa^S^, oxacillin susceptible; BC^b^, MIC of benzalchonium chloride μg/ml.

### Resistance to disinfectants agents

**Figure 1:**
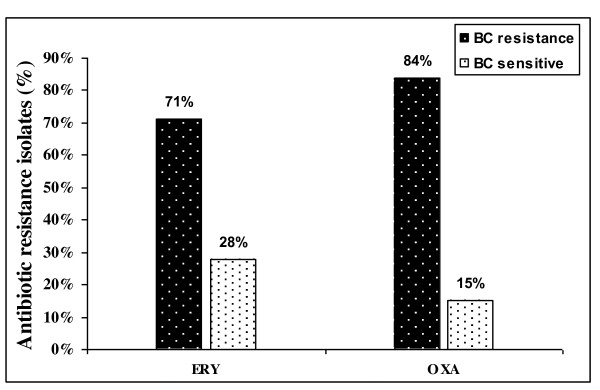
**Percentages of antibiotic-resistance Staphylococci isolates among BC-resistance and BC-sensitive isolates**. Antibiotics used are erythromycin (ERY) and oxacillin (OXA).

The 117 staphylococcus isolates were screened for QAC (BC) resistance. The strains were categorized as BC resistant or sensitive according to the BC MICs. Twenty four (20%) isolates were considered BC highly resistant (BC MICs between 16 and 32 μg/ml), 64 (54%) isolates were resistant to BC (BC MICs between 4 and 8 μg/ml), and 28 (23%) strains were sensitive to BC (BC MICs ≤ 2 μg/ml). 117 staphylococci isolates were analyzed for correlation between BC and antibiotic resistance (Figure 1). This analysis showed that the frequency of erythromycin resistance 71% and oxacillin resistance 84% was higher among the BC-resistant strains.

### Multiplex PCR for the detection of genes encoding macrolide resistance

**Figure 2:**
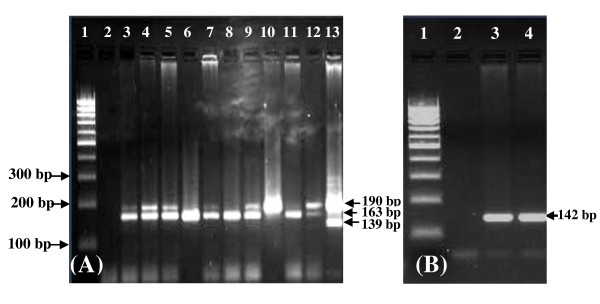
**(A) PCR analysis of erythromycin-resistant determinants: *erm*A (139 bp), *msr*A (163 bp) and *erm*C (190 bp) with clinical strains; source of DNA: 1: 100 bp DNA molecular size marker; 2: negative control; 3: S16; 4: E6; 5: E18; 6: S40; 7: E24; 8: E20; 9: S22; 10: S12; 11: S9; 12: S21 and 13: E15**. (B) Agarose gel electrophoresis of PCR amplicon of erythromycin-resistant *erm*B gene (142 bp) obtained with DNA of clinical strains; 1: 100 bp DNA molecular size marker; 2: negative control; 3: E10 and 4: E11.

In this study we found that the incidence of the three erythromycin ribosomal methylase genes tested was 9 (7.7%) strains of *S. aureus *contained *erm*A, 16 (13.7%) strains harbored *erm*B gene and 7 (6%) strains were positive for *ermC *in the total of 117 isolated strains (Figure 2A, B). Furthermore among the CoNS 11 (9.4%) strains contained *erm*A. In addition, on the total of 117 isolated strains 13 CoNS strains carried the *erm*B gene, while 32 (27.3%) were positive for *erm*C. The *msrA *gene was present in 12 (10.2%) of *S. aureus *strains and in 48 (41%) CoNS strains (Figure 2A). In contrast, the *mef *gene was absent in all the staphylococcal strains tested. Sixteen strains of *S. epidermidis*, eight strains of *S. aureus *and eighteen strains of CoNS were positive for the *mec*A gene, yet were susceptible to oxacillin. Furthermore, only nine strains of CoNS (7.7%) and nineteen strains (16.2%) of *S. aureus *were susceptible to erythromycin, those isolated did not contain any of the erythromycin-resistance genes tested. However, in five *S. epidermidis *strains (E7, E20, S23, S27, and S40), and in ten *S. aureus *stains, the *erm*A and *erm*C genes were not detected, although it was resistant to erythromycin (Table 2 and 3).

### PCR-RFLP detection of *qac*A, *qac*B, and *qac*C genes

**Figure 3:**
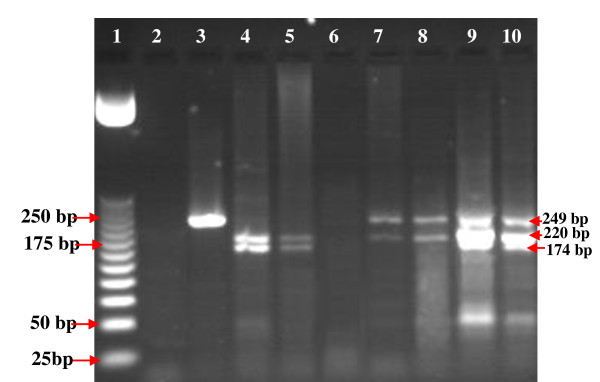
**PCR-RFLP analysis for the detection of *qac*A (220 bp), *qac*B (174 bp), and *qac*C (249 bp) genes in *S.aureus*; 1: 25 bp DNA molecular size marker; 2: negative control; 3: Sa1; 4: Sa5; 5: Sa10; 6: Sa13; 7: Sa19; 8: Sa20; 9: Sa22 and 10: Sa23**.

Among the tested *S. aureus *two strains (Sa22 and Sa23) carried the three *qac *genes (*qac*A, *qac*B and *qac*C) (Figure 3.). While, nine CoNS strains, contained the three *qac *genes (*qac*A, *qac*B and *qac*C). In addition, *qac*C was the most present (35%), followed by *qac*A (24%), and *qac*B (15.4%) in the total of 117 isolated strains (Table 2 and 3).

**Figure 4:**
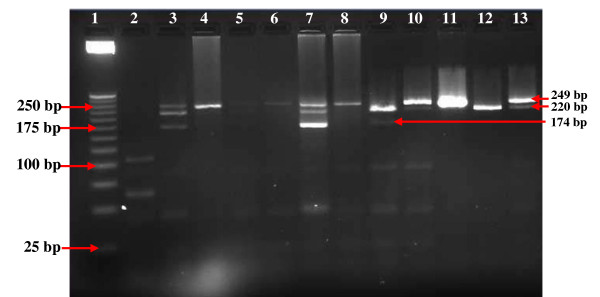
**PCR-RFLP analysis for the detection of *qac*A (220 bp), *qac*B (174 bp), and *qac*C (249 bp) in CoNS; 1: 25 bp DNA molecular size marker; 2: negative control; 3: S9; 4: E15; 5: E13; 6: E6; 7: S18; 8: E7; 9: S13; 10: S19; 11: S11; 12: S45 and 13: S39**.

*Qac*-resistant genes (*qac*A) were identified in (5.12%) followed by *qac*C (4.27%) and *qac*B (3.41%) in the isolated *S. aureus *strains, while 39 (33%) strains were *qac*-negative in the total of 117 isolated strains (Figure 4.). On the other hand, among the 32 *S. epidermidis *strains under study, five were *qac*A+, two were *qac*B+, and 16 were *qac*C+, while 29 strains were *qac*-negative (Table 2 and 3). The three *Qac*-resistant genes (*qac*A, *qac*B, and *qac*C) were identified in 9.4% of the total isolated strains. Nine strains (7.7%) of six different CoNS species harboured the three *qac*-resistant genes.

## Discussion

Recently, the increasing numbers of device-related infections associated with methicillin-resistant staphylococci have raised awareness toward the need for alternative agents to prevent these infections. *S. epidermidis *species represent the CoNS most recovered from clinical specimens [3]. Among the 71 CoNS isolated in this study, the most prevalent were *S. epidermidis *(n = 32), *S. hominis *(n = 10), and *S. haemolyticus *(n = 9). The antibiotic susceptibility of the 117 staphylococal (*S.aureus *and CoNS) isolated in this study confirmed the multi-resistance of these strains toward the 18 antibiotics cited previously. Oxacillin resistance occurred in 7.7% of *S. aureus *and in 20.5% of CoNS tested by ATB Staph.

Erythromycin resistance in staphylococci is predominantly mediated by erythromycin-resistant methylase encoded by *erm *genes [27]. The inducible gene *ermA *is found on the transposon Tn*554 *and has a single specific site for insertion into the *S. aureus *chromosome [28]. The *ermB *gene is found on the transposon Tn*551 *of a penicillinase plasmid [29]. The *ermC *gene is responsible for constitutive or inducible resistance to erythromycin and is generally located on small plasmids [5,27,30]. On the other hand, the investigation of the prevalence of *erm*A, *erm*B, *erm*C and *msr*A genes in Staphylococci showed that only 28 strains of *S. aureus *(n = 46) and CoNS (n = 71) were found to be susceptible to erythromycin, yet contained erythromycin-resistant gene, and 44 strains (13 *S.aureus *and 31 CoNS) have at least one of the four genes (*ermA*, *ermB*, *ermC *and *msrA*) and were susceptible to erythromycin. Similarly, Sekiguchi et al. [26] found discordance among phenotypic susceptibility and the presence of *erm *genes. They stated that this discordance might be due to a mutation in the coding or promoter region of the PCR-detected genes. We noted also that eight strains of *S.aureus *and CoNS strains were found to be resistant to erythromycin but did not carry any erythromycin resistance gene (Table 1 and 2). This result may be explained by the location of these genes in small plasmids, which were occasionally lost. Fluit et al. [31] demonstrated that the *erm*C gene responsible for erythromycin resistance is located on a small plasmid. In this study, the incidences of *ermA *in erythromycin-resistant staphylococci were 7.7% for *S. aureus *and 3.4% for *S. epidermidis*. These findings are in disagreement with the study by Eady et al. [5] conducted with coagulase-negative staphylococci (CoNS) in the United Kingdom in which an incidence of 5.9% for *ermA *was reported. In a study performed in Denmark, 16% of *S. aureus *strains were carrying *ermA*, while only 3% of CoNS strains had this gene [30]. Regarding *erm*B, we found that this gene was more frequently encountered than *erm*A in erythromycin-resistant staphylococci with 24.8% (13.7% for *S.aureus *and 11% for CoNS) of total strains carrying *erm*B. In the United Kingdom, an incidence of 7.2% for *erm*B in CoNS has been reported [5]. Staphylococcal strains resistant to macrolides and type-B streptogramins frequently harbour *msr*A, which encodes an ATP-dependent efflux pump [5]. Erythromycin resistance may be caused by the *msr*A or *erm*B gene, as previously reported with staphylococci [5]. Our results are similar to those of a recent study investigating a high level of *erm*A and *erm*C genes in CoNS [32].

Staphylococcal multidrug-resistant gene *qac*A is generally mediated by plasmids mediated resistance to various toxic organic cations and ethidium bromide, as well as a number of commonly used antiseptics and disinfectants, such as benzalkonium chloride and chlorhexidine [19]. The *qac*A gene also encodes resistance to both monovalent and divalent organic cations. In addition, *qac*B characteristically differs from *qac*A by conferring lower or no resistance to divalent organic cations [33]. Some investigations have implied that there is disinfectant cross-resistance with antibiotics [34,35].

Among the tested bacteria, 2 strains of *S.aureus *and 9 strains of CoNS carried the three *qac *genes (*qac*A, *qac*B, and *qac*C), as shown in Table 1 and 2. *Qac*C gene was the most present in the 117 isolated strains (35%), followed by *qac*A (24%), and *qac*B (15. 4%). Of the 117 isolates investigated in this study, 74% were phenotypically resistant to BC. 56% of the BC-resistant staphylococcus isolates have at least one of the three resistant disinfectants genes (*qacA/B *and *qacC*). Previous investigators have reported a similar distribution of these three *qac *resistance genes in clinical *S. aureus *and CoNS [36,15]. Little is known about the occurrence and possible genetic linkage of *qac *and antibiotic resistance in staphylococci. Interestingly, we observed that staphylococci resistant to BC were generally more often resistant to antibiotics (Ery and Oxa) than BC-sensitive isolates (Figure 1).

Furthermore, among the nine MRSA isolates, two strains (Sa22 and Sa23) were multi-resistant to antibiotic and harboured the *qac *genes. MRSA isolates resistant to antiseptics and disinfectants have been reported in Australia and in United Kingdom in the last decade [8]. Sekiguchi et al., [26] have found that among the 65 MRSA isolates 32 (49.23%) were positive for *qac*A, while one isolate was positive for *qac*B and seven MRSA 10% were positive for *qac*C. Three strains of *S. hemolyticus *harboured *qac*A and *qac*B genes. In a recent study, *S. hemolyticus *isolates were shown to contain both *qac*A and *qac*B genes [37]. *Qac*A/B genes are typically located on a transposon of transmissible multidrug-resistant plasmids, such as pSK1 [33]. Staphylococci resistant genes to quaternary ammonium compounds (QACs) have been detected in clinical coagulase-negative staphylococci [36]. QAC resistant *Staphylococcus *spp. hosting *qac*A/*qac*B and *smr *have been isolated from different environments [38]. Results from a recent study in Norway suggest that *qac*-resistant genes are common in human clinical staphylococci and that a direct link between resistance to QACs and resistance to penicillin occurs in clinical isolates of human and animal origin as well as in food-related staphylococci [11,22]. Noguchi et al. [16] reported that when the antiseptic susceptibility and the distribution of antiseptic-resistant genes of MRSA isolated in Japan in 1992 were studied, *qac*A/B were detected in 10.2% (10/98). However, seven years later, *qac*A/B genes were detected in 47.9% (198/413) in MRSA isolates in Japan.

## Conclusion

It appears that the widespread distribution of staphylococci carrying macrolides and *qac*-resistant genes found in dialysis biomaterial collected in Tunisia may be due to the transfer of resistance plasmids among species and strains, thereby contributing to the dissemination of staphylococcal resistance. Therefore, a closer investigation of antibiotics and antiseptic-resistant CoNS may provide crucial information on the control of nosocomial infections.

## Competing interests

The authors declare that they have no competing interests.

## Authors' contributions

TZ was the primary author of the manuscript, assisted in samples collection, antimicrobial susceptibility, detection of resistance genes and assisted in minimum inhibition concentration determination of BC. BK contributed in minimum inhibition concentration determination, assisted in detection of resistance genes and helped in the writing of the manuscript. HM participated in detection of resistance genes, data acquisition and contributed in writing of the manuscript. AB provided funding, supervised the study, and helped to finalize the manuscript.

All authors read and approved the final manuscript
